# Core taxa drive microeukaryotic community stability of a deep subtropical reservoir after complete mixing

**DOI:** 10.1111/1758-2229.13196

**Published:** 2023-09-09

**Authors:** Yuanyuan Xue, Huihuang Chen, Peng Xiao, Lei Jin, Ramiro Logares, Jun Yang

**Affiliations:** ^1^ Aquatic EcoHealth Group, Fujian Key Laboratory of Watershed Ecology, Key Laboratory of Urban Environment and Health, Institute of Urban Environment Chinese Academy of Sciences Xiamen China; ^2^ University of Chinese Academy of Sciences Beijing China; ^3^ Institute of Marine Sciences (ICM), CSIC Barcelona Spain

## Abstract

Microeukaryotes are key for predicting the change of ecosystem processes in the face of a disturbance. However, their vertical responses to multiple interconnected factors caused by water mixing remain unknown. Here, we conducted a 12‐month high‐frequency study to compare the impacts of mixing disturbances on microeukaryotic community structure and stability over different depths in a stratified reservoir. We demonstrate that core and satellite microeukaryotic compositions and interactions in surface waters were not resistant to water mixing, but significantly recovered. This was because the water temperature rebounded to the pre‐mixing level. Core microeukaryotes maintained community stability in surface waters with high recovery capacity after water mixing. In contrast, the changes in water temperature, chlorophyll‐*a*, and nutrients resulted in steep and prolonged variations in the bottom core and satellite microeukaryotic compositions and interactions. Under low environmental fluctuation, the recovery of microbial communities did not affect nutrient cycling in surface waters. Under high environmental fluctuation, core and satellite microeukaryotic compositions in bottom waters were significantly correlated with the multi‐nutrient cycling index. Our findings shed light on different mechanisms of plankton community resilience in reservoir ecosystems to a major disturbance over depths, highlighting the role of bottom microeukaryotes in nutrient cycling.

## Graphical abstract

In this work, we investigated the microeukaryotic resilience to complete water mixing in a subtropical reservoir and its feedback to nutrient cycling by characterizing core and satellite community compositions in surface and bottom waters, respectively, using 12‐month high‐frequency (every 10 days) observations. Our new findings are: (1) surface microeukaryotic communities are more resilient after water mixing; (2) bottom microeukaryotic communities cannot return to their pre‐mixing state; (3) core taxa drove the community stability and nutrient cycling in both surface and bottom waters; (4) satellite taxa only contributed to nutrient cycling in bottom waters; (5) water temperature was the major factor affecting core and satellite community changes. Our study significantly enhances our understanding of microeukaryotic community responses to water mixing disturbances in deep subtropical reservoirs by addressing existing knowledge gaps. Furthermore, it offers valuable insights into the depth‐dependent mechanisms underlying community compositional resilience in reservoir ecosystems when faced with major disturbances.

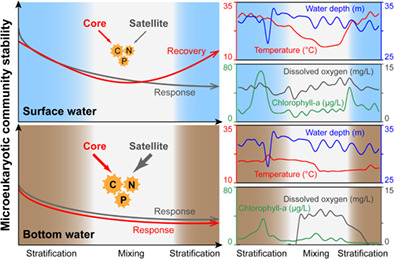

## INTRODUCTION

Inland freshwater ecosystems encounter multiple environmental perturbations under climate‐change scenarios, such as water mixing, extreme warming, and low oxygen (Carey et al., 2022; Woolway et al., 2020; Yang et al., 2021). Microeukaryotes are important for trophic transfer, such as primary production and nutrient cycling, impacting ecosystem services (Hernández‐León et al., 2020; Singer et al., 2021), and maintaining community stability (Liu et al., 2015). Improving our understanding of the factors that determine their community turnover at relevant timescales is important for predicting the recovery of aquatic ecosystems in response to changing environments, especially in reservoir ecosystems. Reservoirs are highly dynamic environments that are influenced by climate change and human activities (Yang et al., 2021). As a result, microbes are temporally variable in these systems, driven by environmental heterogeneity, which can include changes in temperature, nutrients, and oxygen availability (Gao, Chen, et al., 2019).

Subtropical reservoirs typically experience one complete vertical mixing event per year due to low water temperature in winter, further disrupting the physico‐chemical gradients and the different ecological niches for microbes created by thermal stratification (Yu et al., 2014). Therefore, water mixing can be defined as a natural disturbance to microbial communities (Shade et al., 2012). Changes in water column stratification and mixing patterns, on the one hand, influence the nutrient release of the deep lake waters (Vachon et al., 2019). On the other hand, complete water mixing promotes the upwelling of nutrients from deep waters into the surface waters (Gao et al., 2021). This increase in nutrients can cause an increase in phytoplankton biomass or shifts in functional microbial abundance and community structure (French & Petticrew, 2007; Xue et al., 2017). Afterward, an increased temperature from the water mixing to stratification periods can affect zooplankton (e.g., body size and community trophic structure) (Gao, Chen, et al., 2019). Thus, reservoir mixing and microbial communities together create an interesting model system for understanding in situ community stability in a changing environment.

Community stability through periods of water mixing is a topic with many contradictory results in previous studies. In marine ecosystems, the microeukaryotic assemblage at 5 m was temporally more dynamic than deeper assemblages following the seasonal deepening of the mixed layer and stratification of the water column, while bacterial community structure showed seasonal patterns near the surface and bottom of the water column, but not at intermediate depths (Cram et al., 2015; Kim et al., 2014). Shade et al. (2012) demonstrated that bacterial communities returned to their pre‐mixing state in both surface and deep waters after a whole‐lake mixing disturbance, but the surface bacterial communities recovered more quickly. Previous studies mostly focused on the effects of natural water mixing or water mass mixing induced by typhoons on nutrient cycling or phytoplankton bloom in reservoirs (Liu et al., 2012; Yang et al., 2021). However, the effect of water mixing on the microeukaryotic community stability across different water depths remains unclear in reservoirs.

Microbial communities can be divided into broadly distributed core taxa and narrowly distributed satellite taxa in natural ecosystems across space and time (Magurran, 2007). Therefore, the core species represent a small fraction of taxonomic diversity with high abundances, while satellite species contribute to a large reservoir of microbial diversity and occur in low abundance. Core and satellite species show differences in response to environmental changes, for example, Mo et al. (2021) found that core co‐occurrence networks exhibited strong resistance as salinity increased, while satellite networks are prone to be affected by slight disturbances of salinity. Compared with satellite taxa, core taxa had wider niche breadths and could adapt to a wide range of environmental niches (Mo et al., 2021). Core taxa can play key roles in enhancing the community resistance of soil microbiome (Jiao et al., 2022b). Satellite taxa, analogous to rare taxa, may be more influenced by environmental changes and maintain community stability (Wang et al., 2023; Xiong et al., 2021). These results showed that core and satellite species play different roles in characterizing community structure. Due to climate change, increasing surface water temperature has led to mixing‐regime alterations in lake systems (Woolway et al., 2020). Thus, it is imperative to understand the effect of water mixing on the shift and stability of core and satellite species, gaining a more comprehensive insight into community recovery capability.

The resilience of the microbial communities is highly dependent on the microbial community turnover (Huang et al., 2020; Wu et al., 2021), further influencing ecosystem functioning. A previous study found that the core microbiota exhibited significantly positive relationships with the functional stability of the soil microbiome, highlighting the importance of the core microbiota in functional stability maintenance (Jiao et al., 2022a). Wang et al. (2023) also demonstrated that the core microbiome may potentially provide critical functions to the arbuscular mycorrhizal fungi by promoting organic phosphorus mineralization. However, the beta diversity of satellite communities was more significantly related to multi‐nutrient cycling than core communities in a subtropical urban reservoir (Mo et al., 2021). These results imply that core and satellite taxa play different roles in maintaining ecological function in different ecosystems. Gao et al. (2021) found that nutrients and particulate organic matter in the bottom waters had a more prolonged response to typhoon‐induced mixing disturbances than those in the surface waters in our studied deep reservoir. However, we still do not comprehend the contributions of core and satellite species to driving nutrient cycling in deep reservoirs.

**FIGURE 1:**
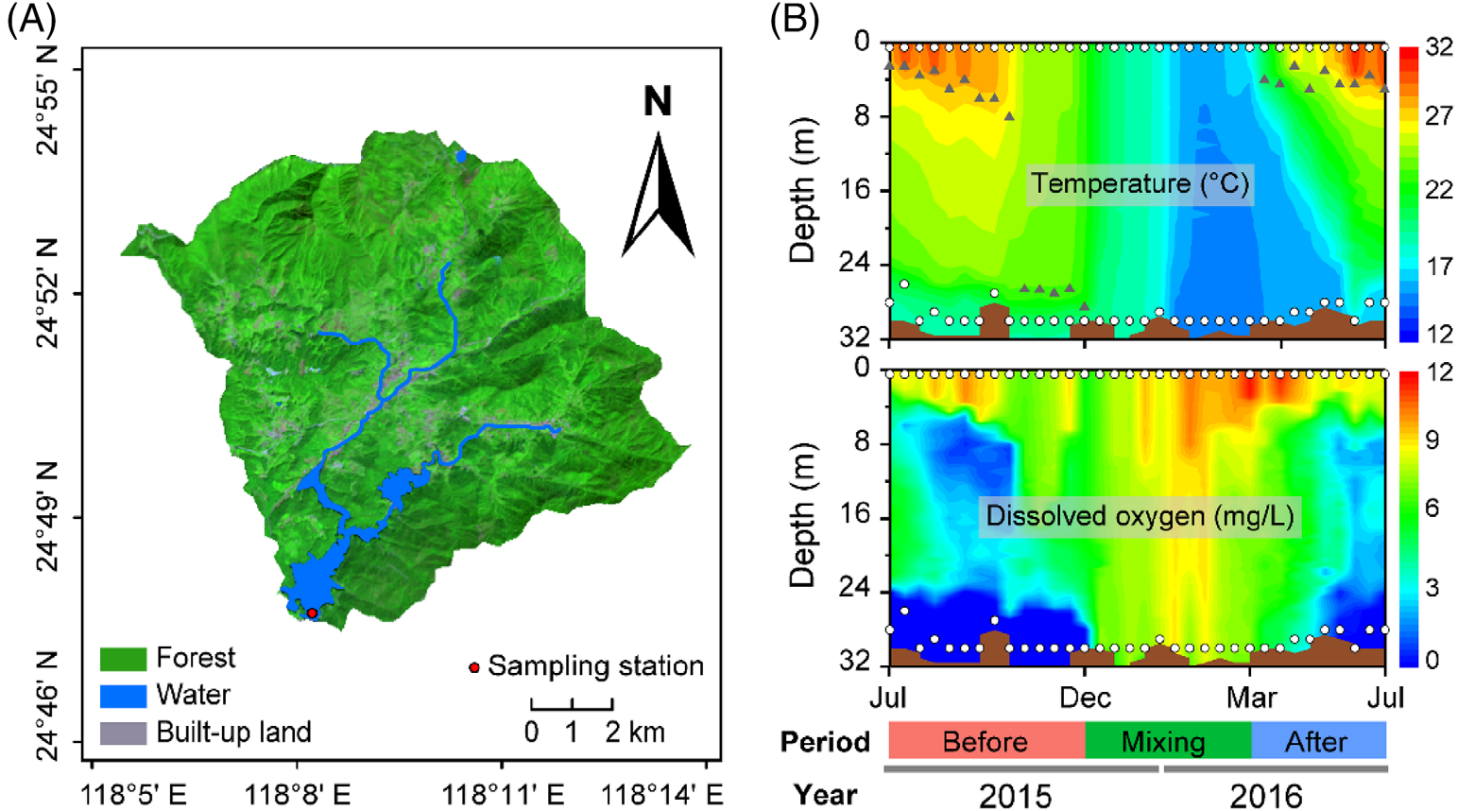
Sampling site and sampling depths in Tingxi Reservoir. Sampling station in Tingxi Reservoir (A). Forest covers more than 85% of the Tingxi watershed. Depth‐time profiles of water temperature and dissolved oxygen from July 2015 to July 2016 in Tingxi Reservoir (B). White dots represent sampling depths from stratification (14 sampling visits) to complete mixing (11 sampling visits) to re‐stratification (9 sampling visits) periods. Grey triangles represent the variations in thermocline depth. Before indicates before the complete mixing (stratification) period; mixing indicates the complete mixing period; after indicates after the complete mixing (re‐stratification) period.

Here, we investigate the microeukaryotic resilience to water mixing in a subtropical deep reservoir (i.e., Tingxi Reservoir, Figure 1A) and its feedback to nutrient cycling by characterizing the temporal dynamics of core and satellite microeukaryotic communities at both surface and bottom waters. The objectives of our study were (1) to compare the resilience of core and satellite subcommunities to water mixing between surface and bottom waters; (2) to identify the major environmental factors driving the changes in core and satellite subcommunities' stability; (3) to compare the ecological roles of the core and satellite taxa in determining the community stability and function along water depth.

## EXPERIMENTAL PROCEDURES

### Sample collection

The field sampling was conducted near the dam of the Tingxi Reservoir (24°48′ N, 118°08′ E), which was located in Xiamen City, Fujian Province, Southeast China (Figure 1A). Surface (0.5 m) and bottom (2 m above the bottom sediment) water samples were collected nearly every 10 days from July 2015 to July 2016 (Figure 1B). During the sampling, the water column of the Tingxi Reservoir (average depth of 31.7 m) changed from stratification to complete mixing to re‐stratification stages. We defined the stratification and mixing periods based on the occurrence of a thermocline in the water column, and water temperature profiles were used to determine the thermocline (Figure 1B). The water layer with more than the vertical gradient of 0.2°C/m was defined as the thermocline depth (Zhang et al., 2015). Based on this criterion value, three well‐defined periods are used for each water depth: the stratification period before mixing (July 2015–December 2015, *n* = 14), the complete water mixing period (December 2015–March 2016, *n* = 11), and the re‐stratification period after mixing (April 2016–July 2016, *n* = 9). In total, 34 surface and 34 bottom water samples were obtained, respectively. For each sample, 800–1000 mL water was pre‐filtered through a 200‐μm mesh to remove larger metazoans and other particles, and then through 0.22‐μm polycarbonate filters to collect the microeukaryotic cells (47 mm, Millipore, Billerica, MA, USA). The filters were stored at −80°C until DNA extraction. Environmental parameters of the water were measured, and detailed descriptions were shown in Supplementary Methods.

### DNA extraction, sequence processing, and bioinformatics analysis

Filters containing 0.2–200 μm plankton fractions were cut into small species, and DNA was extracted using the FastDNA spin Kit (MP Biomedicals, Santa Ana, CA, USA). Microeukaryotic community composition and diversity were assessed by amplifying the V9 region of the 18S rRNA gene using primers 1380F (5′‐CCCTGCCHTTTGTACACAC‐3′) and 1510R (5′‐CCTTCYGCAGGTTCACCTAC‐3′) with barcodes (Amaral‐Zettler et al., 2009). Each DNA sample and negative control were run in triplicate. The 30‐μL PCR mixture included 15 μL of Phusion R High‐Fidelity PCR Master Mix (New England Biolabs, Beverly, MA, USA), 0.2 μM of forward and reverse primers, and 10 ng template DNA (Liu et al., 2019). The PCR reaction conditions included an initial denaturation at 98°C for 1 min, followed by 30 cycles of 10 s at 98°C, 30 s at 50°C, 30 s at 72°C and a final extension at 72°C for 5 min (Liu et al., 2019). The gel‐purified PCR products from triplicate reactions per sample were processed to prepare libraries and sequenced on an Illumina HiSeq platform (2 × 150 bp) (Illumina, Inc., San Diego, CA, USA).

Quality check and merge of paired‐end reads were conducted using MOTHUR (Mo et al., 2021), and sequences were then collapsed into unique sequences using VSEARCH v2.14.1 with the option ‘minuniquesize 8’ (Rognes et al., 2016). Chimeras were removed, and sequences were clustered into operational taxonomic units (OTUs) at 97% similarity and mapped back to the original filtered sequences to obtain the total number of sequences for each sample using the unoise3 algorithm in USEARCH 11 (Edgar & Flyvbjerg, 2015). Representative sequences of microeukaryotic OTUs were taxonomically assigned against the Protist Ribosomal Reference (PR2 V. 4.10.0) database (Guillou et al., 2013). To minimize sequencing errors in extremely rare taxa, we deleted OTUs with less than 10 sequences. Microeukaryotic OTU abundance tables were resampled to the minimum of 121,243 sequences per sample using MOTHUR v1.39.5 (Schloss et al., 2009).

Considering that the microeukaryotic OTUs defined at the 97% similarity level with the unoise3 algorithm is not a specific and accurate estimation of the species level diversity, we also defined amplicon sequence variants (ASVs) using the DADA2 approach following a previous study (Abdullah Al et al., 2022). Our study focused on the comparisons of microeukaryotic communities before and after water mixing. Thus, we compared the beta‐diversity analysis of microeukaryotic plankton using ASVs and OTUs and assessed simply whether our results were biased by the OTUs.

### Definition of core and satellite taxa

Microbial communities can be partitioned into core and satellite taxa according to their occurrence frequency and abundance (Ju & Zhang, 2015). In this study, core and satellite OTUs were separately defined for surface and bottom waters (Figure S1). In each water depth, core taxa were defined as the OTUs with an occurrence frequency of ≥75% within all samples, whereas satellite taxa were defined as having an occurrence frequency of <50% within all samples (Mo et al., 2021). Overall, the mean relative abundance and occurrence frequency of OTUs had significant positive relations (Figure S1A). This indicated that core OTUs were generally more abundant than satellite OTUs, although the core taxa included a much lower number of OTUs (Table S1).

To deeply explore the contributions of core and satellite taxa to the abundances of microbial communities, the overall OTUs for each water depth were categorized into abundant and rare taxa based on their relative abundance by setting 1% as abundant OTUs and 0.01% as rare OTUs, respectively (Xue et al., 2018). Core taxa mainly included always abundant taxa (AAT), conditionally abundant taxa (CAT), conditionally rare and abundant taxa (CRAT), and conditionally rare taxa (CRT). Satellite taxa included CRT and always rare taxa (ART).

### Microeukaryotic 18S rRNA gene abundance

The copies of the microeukaryotic 18S rRNA gene were estimated using a LightCycler 480 Real‐Time PCR System (Roche Diagnostics Ltd., Rotkreuz, Switzerland). Amplification systems and thermal cycling reaction conditions were carried out according to our previous study (Mo et al., 2021). All amplifications were performed in triplicate, and we set negative controls throughout the experiment. Standard curves were constructed using a 10‐fold gradient dilution of plasmids containing a fragment of an insert of the 18S rRNA gene of *Cryptomonas pyrenoidifera* (Cryptophyta). The amplification efficiency of standard curves ranged from 90% to 110% in this study. The absolute abundance of each core and satellite OTU in each sample was calculated by the total copy numbers of the 18S rRNA gene measured by qPCR multiplied by the relative abundances of each core or satellite OTU.

### Core and satellite community stability

We conducted a 5‐time moving window analysis to illustrate the temporal stability of core and satellite microeukaryotic communities and their response to mixing disturbance using the ‘ggplot2’ R package (Wickham et al., 2016). A total of 34 samples at each water depth were organized into the time series as 30 overlapping 5‐sample groups. Therefore, the first window corresponds to samples 1–5, the second window to samples 2–6, and window 11 to samples 11–15. Window 11 is thus the first community subset including one sample of the complete mixing period. We quantified community stability as the inverse of the coefficient of variation (1/CV) of core and satellite absolute abundance (Chang et al., 2020). 1/CV can measure the degree of constancy in a variable relative to its mean (Tilman et al., 2006), and 1/CV is computed as *μ*/*σ*, where *μ* and *σ* indicated the mean and standard deviation of core and satellite absolute abundance, respectively. The 30 moving windows were divided into three different groups based on the occurrence of complete mixing samples: Group 1 only includes the community subset before the complete mixing period (*n* = 10); Group 2 includes the community subset of the following mixing period (*n* = 15); Group 3 only includes community subset after the complete mixing period (*n* = 5). Finally, we assessed the contributions of core and satellite community stability to entire community stability by calculating their relationships using the ‘ggplot2’ R package (Wickham et al., 2016).

### Multi‐nutrient cycling index

Ecosystems simultaneously provide multiple ecosystem functions and services (Manning et al., 2018). Multiple nutrient cycling is an important ecosystem process (Delgado‐Baquerizo et al., 2017). We constructed a multi‐nutrient cycling (MNC) index with reference to recent publications (Gao et al., 2021; Jiao et al., 2018; Mo et al., 2022) using total carbon, total organic carbon, total nitrogen, ammonium nitrogen, nitrate nitrogen, nitrite nitrogen, total phosphorus, and phosphate phosphorus. These nutrient properties are essential components of microbial biomass, often limit primary production, and influence biogeochemical processes (Stein, 2020; Trommer et al., 2019). To quantify the MNC for each sample, each nutrient property was first log(*x* + 1)‐transformed, and then *Z*‐score transformation. Finally, we averaged these standardized datasets of all individual nutrients to get the MNC for every sample.

### Other statistical analyses

The differences in 18S rRNA gene abundance among periods were calculated using the Kruskal–Wallis test. To look for evidence of community recovery under the water mixing, first, significant differences in microeukaryotic beta diversity were calculated between surface and bottom waters and among different periods via nonmetric multidimensional scaling (NMDS) and analysis of similarities (ANOSIM) using the ‘vegan’ and ‘ggplot2’ R packages (Wickham et al., 2016). Second, time‐lag analyses were performed to analyse the dynamics of Bray–Curtis dissimilarity, and the time difference (month) was then plotted against the community dissimilarity using the ‘ggplot2’ R package (Wickham et al., 2016). Third, the difference in Bray–Curtis community dissimilarities between different periods was examined based on the Kruskal–Wallis test using the ‘agricolae’ and ‘stats’ R packages. We used similarity percentage (SIMPER) analysis to measure the contribution of each taxon to community dissimilarity over three periods.

To explore the effect of complete water mixing on network‐level topological properties, co‐occurrence networks of core and satellite microeukaryotic communities were constructed for surface and bottom waters, respectively. To reduce rare OTUs in the dataset, we only kept the shared OTUs among three periods with a relative abundance of ≥0.05%. Networks were constructed based on strong Spearman correlation coefficients of ≥|0.8| and false‐discovery‐rate‐corrected *p* values of <0.01 using the ‘picante’ R package (Jiao et al., 2020). Subnetworks for each sample were generated from the whole network using the subgraph function in the ‘igraph’ R package and the topological properties of subnetworks were estimated, including node number, edge number, average path length (the average network distance between all pairs of nodes), betweenness centralization (potential influence of a particular node on the connections of other nodes), density (intensity of connections among nodes), and modularity (a measure of whether connections tend to occur within or between modules) (Chen et al., 2021; Huo et al., 2023). Kruskal–Wallis test was employed to compare the differences in each topological feature among three different periods using the ‘agricolae’ and ‘stats’ R packages.

Partial least squares path modelling (PLS‐PM) was constructed to identify the direct and indirect links between the drivers and the MNC using the ‘plspm’ package (Sanchez, 2013). Before analysis, we performed a hierarchical cluster analysis to filter the collinearity variables using the ‘Hmisc’ R package (Harrell Jr & Harrell Jr, 2019). On the environmental variables, squared Spearman correlations were calculated among all variables, and one of any two variables that had squared Spearman coefficients ≥0.5 was arbitrarily removed. The number of network properties was few, and we only deleted one of any two variables that had squared Spearman correlation coefficients ≥0.6. The drivers included seven block variables: water temperature (WT), physicochemical factors (Phys), nutrient (Nutr), chlorophyll‐*a* (Chl‐*a*), microeukaryotic NMDS axis 1 score, microeukaryotic NMDS axis 2 score, and microeukaryotic network properties (Net). We did not analyse the relationships between Nutr and MNC. Endogenous latent variables with loading value <0.7 were removed. Model performance was checked using the goodness of fit statistic (GoF) and coefficients of determination (*R* ^2^).

## RESULTS

### Changes in environmental features following water mixing

The decrease in water temperature induced complete water mixing in December 2015. Bottom dissolved oxygen (DO) concentration increased from below the detection limit to a mean of 7.85 mg L^−1^ but then returned to its anoxic conditions before mixing without significant difference after water mixing (Figure S2). Although the surface water temperature significantly decreased during the complete mixing period, it recovered to the pre‐mixing condition without significant differences after water mixing (Figure S2). The concentrations of the bottom ammonium nitrogen (NH_4_^+^) and nitrate nitrogen (NO_3_^−^) decreased and increased during the complete mixing period, respectively. The bottom water temperature, chlorophyll‐*a* (Chl‐*a*), total nitrogen (TN), and total phosphorus (TP) significantly decreased after water mixing.

### Effects of water mixing on microeukaryotic abundances and community compositions

Complete water mixing significantly decreased the absolute abundances of core and satellite microeukaryotes, which only recovered in the surface waters following the water re‐stratification, but not in the bottom waters (Figure S3). The absolute abundances of the core and satellite microeukaryotes were mainly related to water temperature in the surface waters, while the water temperature and Chl‐*a* were mainly related to the absolute abundances of the core and satellite microeukaryotes in the bottom waters (Table S2).

The microeukaryotic communities were structured between two water depths and among three periods based on our NMDS analysis and ANOSIM test (*p* < 0.01, Figure S4). NMDS results of core and satellite ASVs exhibited almost identical patterns as those based on OTUs (Figure S4). Pairwise Bray–Curtis dissimilarities of core or satellite taxa showed striking similarity (*r* > 0.9, *p* < 0.01) between the OTUs and ASVs results (Figure S5). In this study, we only explored the shifts in the microeukaryotic community using OTUs.

**FIGURE 2:**
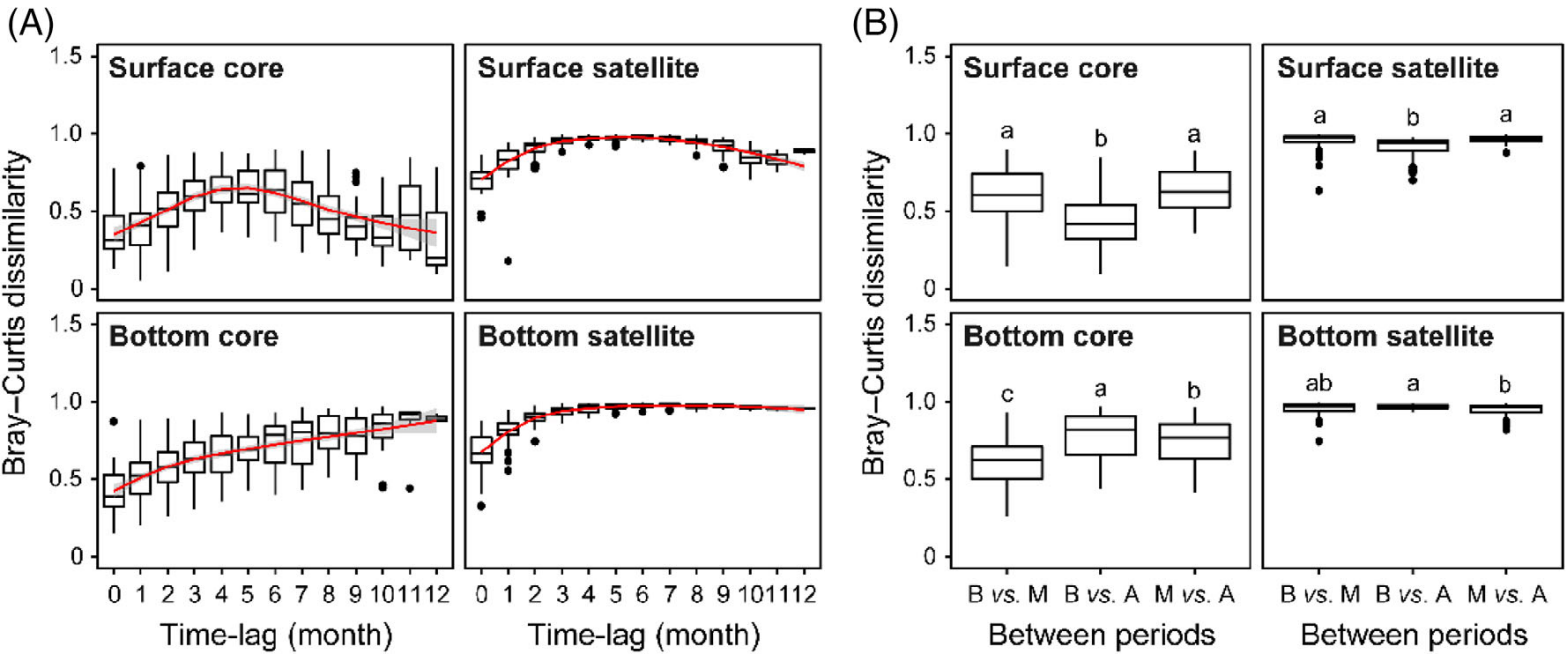
Microbial beta diversity associated with the three different periods (B, M, A periods). Temporal turnover of core and satellite community compositions in surface and bottom waters over time, respectively (A). Differences of pairwise Bray–Curtis dissimilarity of microeukaryotic plankton communities between different periods in surface and bottom waters, respectively (B). B indicates before the complete mixing (stratification) period; M indicates the complete mixing period; A indicates after the complete mixing (re‐stratification) period. Different lowercase letters indicate significant differences based on the Kruskal–Wallis test at *p* < 0.05 level. Boxplots show median (black line), 25th and 75th percentiles (box), and range (whiskers); dots represent outliers.

The dissimilarities of the core and satellite communities increased from the time‐lag 0–5 months and exhibited a sharp decline when time‐lag ≥6 months (Figure 2A). Furthermore, the core and satellite community dissimilarities before versus after mixing were significantly lower in the surface waters than the differences in other pairwise comparisons between any two periods (Figure 2B). These indicated that the core and satellite communities were affected by water mixing and had recovered. In contrast, the core and satellite communities in the bottom waters had not recovered after water mixing, and its average community dissimilarity between samples increased gradually with month intervals (Figure 2A). Furthermore, the highest community dissimilarity before versus after mixing was observed in the bottom waters (Figure 2B).

Few core OTUs predominantly contributed to the dissimilarity of community composition in both surface (58.9%) and bottom (56.9%) waters, of which conditionally abundant taxa (CAT) and conditionally rare and abundant taxa (CRAT) were major contributors to the community rearrangements in both surface and bottom waters (Figure S6B; Table S1). In contrast, a large number of satellite OTUs only explained 21.4% and 24.2% of the total community dissimilarity in surface and bottom waters, respectively (Figure S6B; Table S1). The top five contributors to the core community dissimilarity in the surface waters were Arthropoda (28.3%), Cryptophyceae (7.8%), Katablepharidaceae (4.3%), Spirotrichea (2.4%), and Chrysophyceae (1.9%) (Figure S7). The top five contributors to the core community dissimilarity in the bottom waters were Arthropoda (22.5%), Cryptophyceae (5.7%), Katablepharidaceae (3.5%), Chrysophyceae (2.8%), and Chlorophyceae (2.3%) (Figure S7). The top five contributors to the satellite community dissimilarity in the surface waters were Chrysophyceae (2.0%), Chytridiomycota (2.0%), Dinophyceae (1.5%), Chlorophyceae (0.9%) and Oomycota (0.8%) (Figure S7). The top five contributors to the satellite community dissimilarity in the bottom waters were Chrysophyceae (2.0%), Dinophyceae (1.5%), Euglenozoa (1.3%), Spirotrichea (1.0%) and Arthropoda (0.9%) (Figure S7).

### Effects of water mixing on microeukaryotic community stability

**FIGURE 3:**
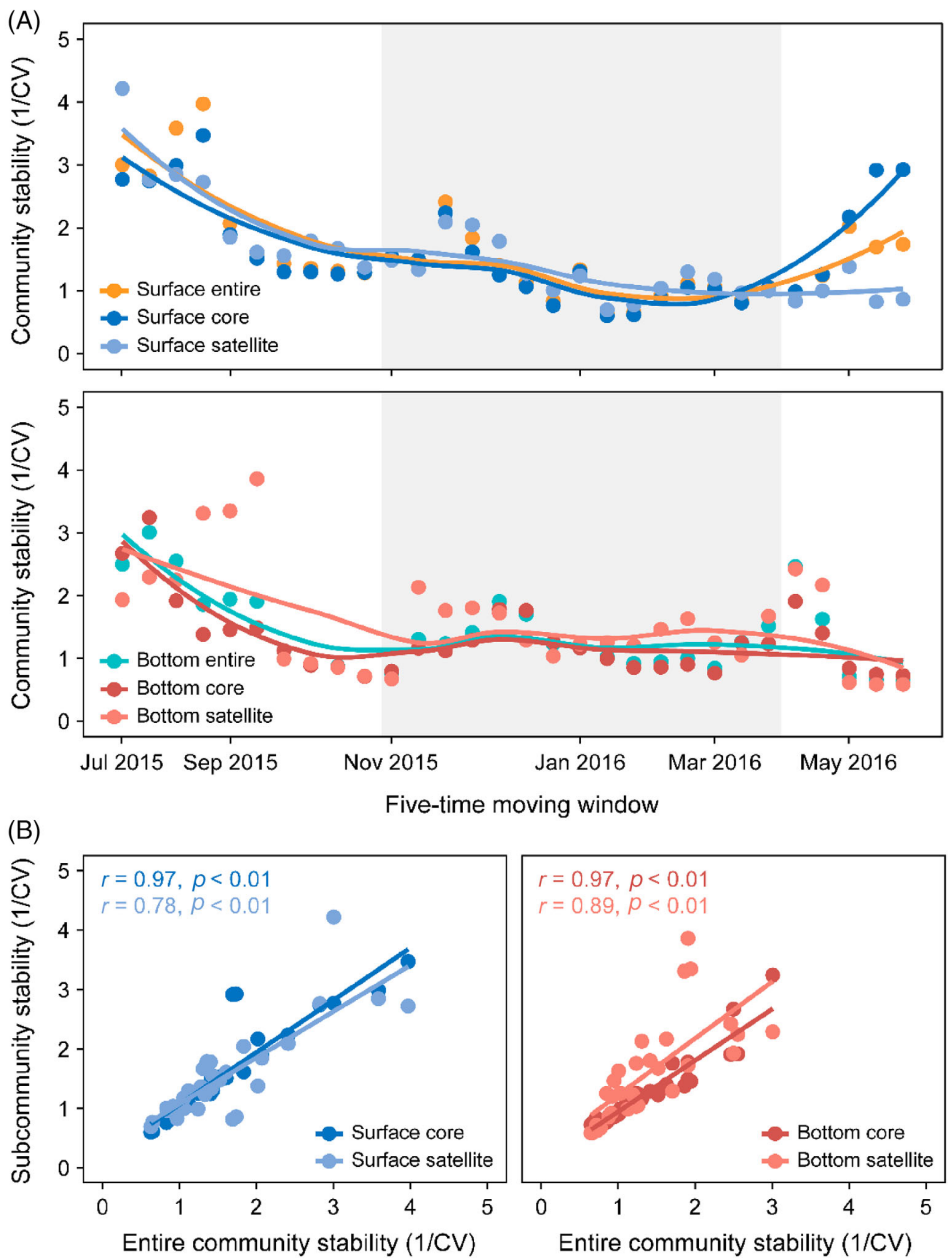
Community stability was quantified over time as the inverse coefficient of variation (1/CV) of microbial absolute abundance. Temporal variability of the entire, core and satellite microeukaryotic community stability in surface and bottom waters over each 5‐time point window (A). Complete water mixing was defined as the disturbance period, and the grey area includes the community subset from disturbance start (window 11) to disappearance (window 25). Window 11 was the first community subset including one sample of the complete mixing period. Relationships between core or satellite community stability and the entire community stability for surface and bottom waters (B).

We quantified the community stability over time using the inverse coefficient of variation (1/CV) of the entire, core, and satellite microeukaryotic absolute abundances (Figure 3). We found that the occurrence of complete mixing perturbation decreased the microeukaryotic community stability in both surface and bottom waters, but they had different patterns. The entire and core community stability significantly decreased during perturbation and increased after perturbation in surface waters, while the satellite community stability gradually decreased (Figure 3A). For bottom waters, the entire, core, and satellite community stability steadily remained at low levels after perturbation (Figure 3A). Although satellite community stability exhibited a significant positive relationship with the entire community stability for surface and bottom waters, core community stability showed a higher correlation with the entire community stability (Figure 3B). This suggested that core community mainly contributed to the overall community stability in surface and bottom waters.

We also compared core and satellite microeukaryotic stability using ASVs and OTUs results. Community stability generated by OTUs for both surface and bottom waters showed a high correlation to the community stability generated by ASVs (Figure S8A). Core community stability had higher correlations with the entire community stability than the satellite community stability using ASVs (Figure S8B).

### Effects of water mixing on microeukaryotic networks

**FIGURE 4:**
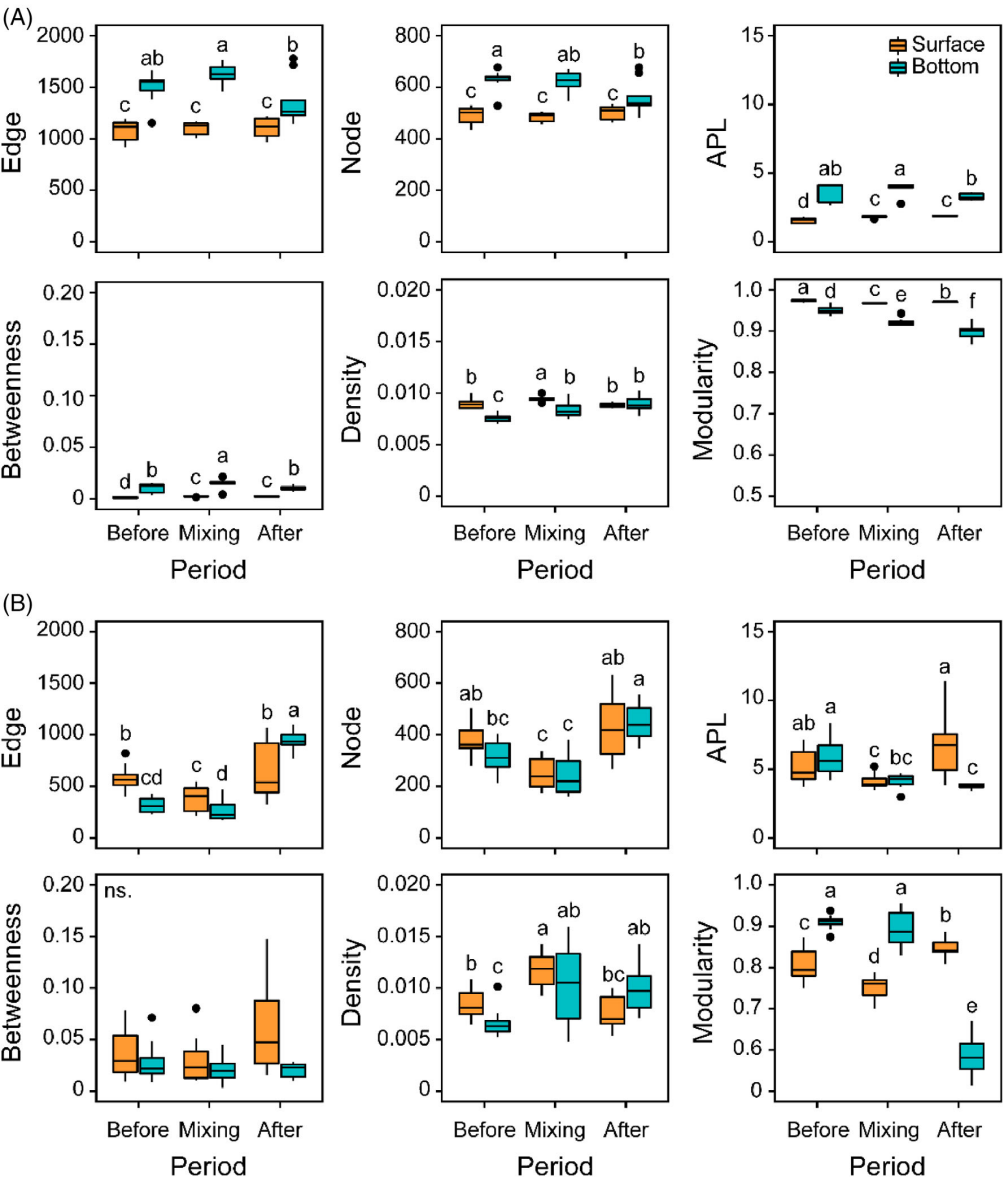
Comparisons of topological parameters of core (A) and satellite (B) microeukaryotic networks among three periods in surface and bottom waters. Before indicates before the complete mixing (stratification) period; mixing indicates the complete mixing period; after indicates after the complete mixing (re‐stratification) period. Different lowercase letters indicate significant differences based on the Kruskal–Wallis test at *p* < 0.05 level. Boxplots show median (black line), 25th and 75th percentiles (box), and range (whiskers); dots represent outliers. APL, average path length; ns., non‐significant differences.

For the surface core networks, water mixing significantly increased network density, which recovered to the level before mixing without significant differences after mixing (Figure 4A). For the bottom core networks, network density significantly increased after the complete mixing, while network edges, nodes, and modularity significantly decreased (Figure 4A). The topological parameters (edges, nodes, average path length, and modularity) of the surface satellite networks significantly decreased from before mixing to the mixing period, and then returned to the pre‐mixing level after mixing (Figure 4B). Network density had the reverse pattern. The topological parameters (edges, nodes, and density) of the bottom satellite networks increased after mixing, while average path length and modularity decreased (Figure 4B).

### Factors associated with microeukaryotic communities and nutrient cycling

**FIGURE 5:**
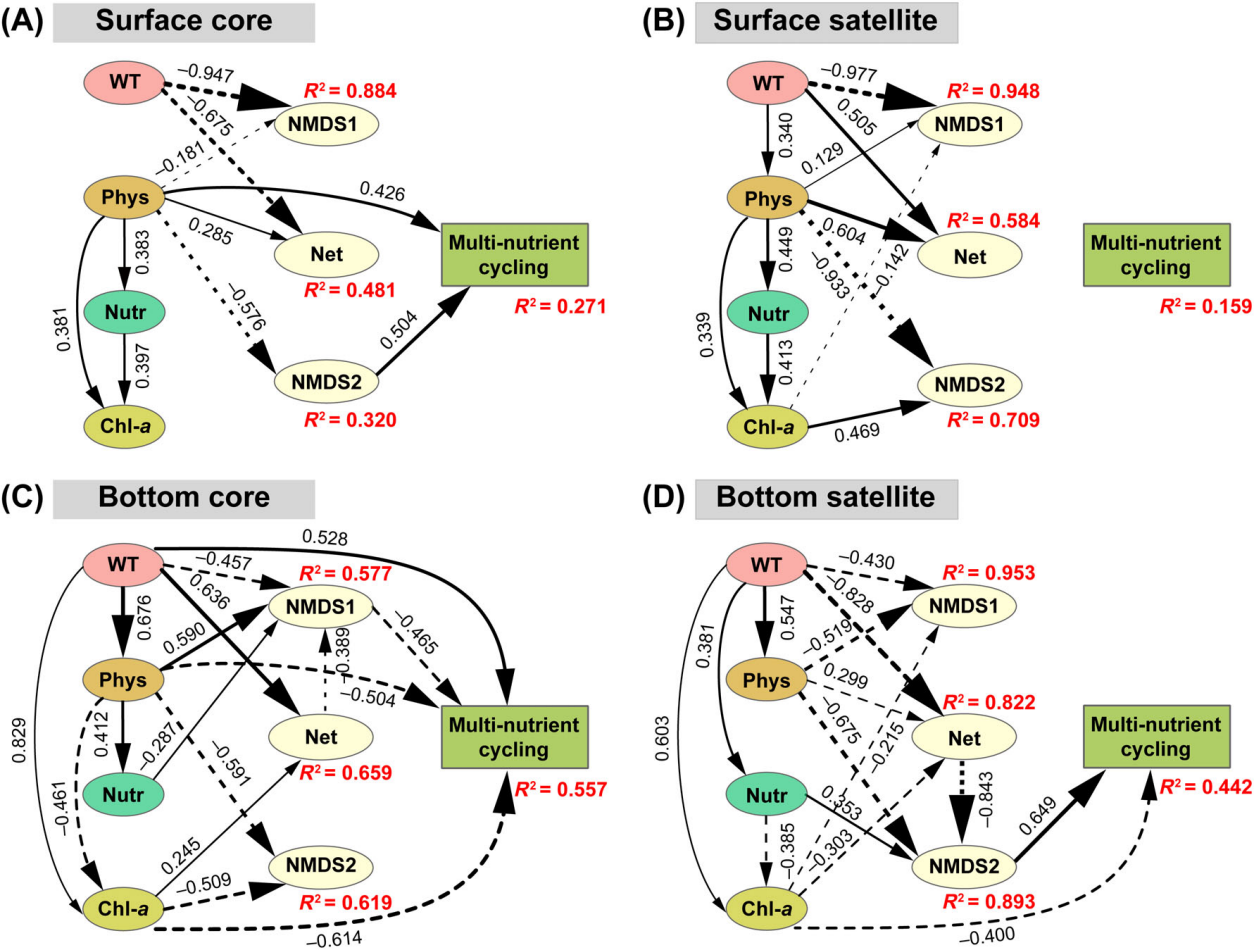
Partial least squares path models (PLS‐PM) showing the relationships between environmental factors, core and satellite microeukaryotic communities (nonmetric multidimensional scaling [NMDS] axes 1 and 2), network properties, and multi‐nutrient cycling index in surface and bottom waters, respectively. (A, B) surface core and satellite taxa; (C, D) bottom core and satellite taxa. Only significant (*p* < 0.1) paths are shown. Solid and dashed lines indicate positive and negative effects, respectively. The thickness of the line indicates the absolute value of the path coefficients. *R* ^2^ denotes the degree explained by their independent latent variables. The goodness‐of‐fit index for (A)–(D) are 0.494, 0.537, 0.571, and 0.599, respectively. Chl‐*a*, chlorophyll‐*a*; Net, network properties; Nutr, nutrient variables; Phys, physicochemical factors; WT, water temperature.

According to PLS‐PM analysis, the water temperature had the largest and most significant direct effect on the core and satellite community compositions (NMDS axis 1) in surface waters (Figure 5A,B). For bottom waters, water temperature, nutrient (e.g., TN), physico‐chemical variables (e.g., DO), Chl‐*a*, and network topological properties had direct and significant effects on the core and satellite community compositions (NMDS axes 1 and 2) (Figure 5C,D). Our PLS‐PM results showed that only the core community was significantly correlated with the MNC index in the surface waters (Figure 5A). Both core and satellite communities were significantly correlated with MNC in the bottom waters, and Chl‐*a* was significantly correlated with MNC (Figure 5C,D). Additionally, water temperature, nutrients, physico‐chemical variables, Chl‐*a*, and network topological properties were potential drivers for MNC via affecting core and satellite communities. Overall, the seven variables can explain larger variations in core and satellite communities in bottom waters than in surface waters (Figure 5).

## DISCUSSION

Our understanding of the responses of microorganisms to disturbances in multiple ecosystems has increased rapidly in recent years (Chen et al., 2022; Delgado‐Baquerizo et al., 2017; Gao, Chen, et al., 2019). However, few studies have investigated the response of core and satellite microeukaryotes to water mixing disturbance in reservoir ecosystems with depth. In this study, we found that the differences in environmental fluctuation strengths drove different community compositions between the surface and bottom waters. Core microeukaryotes played a major role in maintaining community composition and functional stability in reservoirs, whereas satellite taxa only drove stronger potential multi‐nutrient cycling in bottom waters.

### Distinct responses of core and satellite microeukaryotes to water mixing with depth driven by environmental variability

After disturbance occurs, if communities can return to their original state, they could be considered stable, while communities may be constantly changing or shift to an alternative stable state at a given period (Lamothe et al., 2019). Microbial communities in this study followed different succession trajectories with depth, and environmental changes caused by water mixing showed an important relationship with microbial community shift. We found that water temperature was the major contributor to the difference in the core and satellite communities with depth. The recovery of the surface temperature directly promoted the recovery of core and satellite communities after complete water mixing. A decrease in water temperature after water mixing boosted persistent changes in microbial communities in the bottom waters. Water mixing simultaneously decreased the water temperature across the water column (Li et al., 2020), but only surface water temperature returned to its pre‐mixing state in the stratification of the following year. Previous work has demonstrated that water temperature is a main factor affecting the seasonal cycles of the microeukaryotes in surface marine waters (Giner et al., 2019). Meanwhile, the surface core and satellite network properties showed recurrent correlations with water temperature. Previous studies demonstrated that the microbial network properties can be affected by environmental factors and drive the patterns of microbial diversity (Chafee et al., 2018; Gao, Yang, et al., 2019).

Except for water temperature, the changes in other environmental factors driven by water mixing influenced the persistent shifts of the bottom core and satellite communities. First, water mixing decreased the bottom nutrients, further influencing the core and satellite community dynamics. Complete water mixing boosts the upwelling of nutrients from deep waters into the upper waters (Lao et al., 2023). Due to nitrification, ammonium, and nitrate concentrations would be lower and higher in oxic conditions, respectively (Carey et al., 2022). Total nitrogen in the hypolimnion could decrease in oxic conditions, depending on the balance of ammonium and nitrate (Carey et al., 2022). This was consistent with the depleted ammonium concentration and a steep increase in nitrate concentration during water mixing. Second, water mixing exerted a significant effect on the core and satellite community compositions as measured by chlorophyll‐*a*. Lower chlorophyll‐*a* concentration in the mixing period may be correlated with lower particulate organic matter sinking (phytoplankton and dead organic matter) across the water column. This particulate sinking from the upper waters strongly contributed to the community dynamics and biogeochemical processes of the bottom waters (Gao et al., 2021; Mestre et al., 2018). Chlorophyll‐*a* concentration significantly declined in the bottom waters after complete mixing and was significantly related to core and satellite absolute abundances. Altogether, the environmental succession trajectory was not disrupted in the surface waters. In contrast, continuously changing environments (e.g., temperature, nutrients, and chlorophyll‐*a*) disrupted the succession trajectory in the bottom waters, thus promoting the prolonged response of deep microeukaryotes to the complete water mixing event.

### Importance of core taxa to community stability

Different microorganisms exhibit contrasting responses to environmental changes, further leading to different temporal stability of microbial communities (Mo et al., 2021). We found that core rather than satellite microeukaryotes dominated the community stability for both surface and bottom waters, suggesting that the resilience of core taxa was a key determinant of the temporal stability of the microbiome in a changing ecosystem. These core OTUs may have acted as pioneers driving the community succession, which may determine contrasting community patterns across water depths. Results from Shade et al. (2010) also supported this view that microorganisms (generalists) in both the upper and deep environments were important for community re‐assembly after water mixing. Previous studies demonstrated that core species played a crucial role in maintaining the temporal stability of microbiomes in a given host species or agricultural soil environment (Björk et al., 2018; Jiao et al., 2019). This can be explained by different life strategies, for example, abundant taxa normally occupy more broader niches, more competitively utilize an array of resources, and more effectively adapt to the environment in comparison with rare taxa (Barberán et al., 2014; Jousset et al., 2017). Dominant OTU populations were effective trackers of environmental conditions, in agreement with previous results (Bier et al., 2022).

A previous study showed that subsets of a few hundred dominant OTUs can be highly representative of entire communities, and these dominant OTUs had a high abundance (Gschwend et al., 2021), which supports our results. In our study, few core OTUs had high abundances, and they were composed of conditionally abundant, conditionally rare, and conditionally abundant and rare OTUs (Figure S6A). These taxa had higher cumulative contributions to entire community dissimilarity due to transitions in abundance from abundant to rare triggered by environmental cues (Giner et al., 2019; Shade et al., 2014). Satellite OTUs were mainly composed of conditionally rare and always rare OTUs with low abundances (Figure S6A). They were sensitive to environmental changes and contributed to community shifts to a certain extent (Figure S6B). Permanently rare OTUs steadily remain in low abundance and randomly occur due to stochastic processes, or sequence‐dependent errors (Jia et al., 2018, 2022). Thus, they have little impact on the shift of community structure compared to core taxa in reservoirs.

### Ecological roles of the core and satellite microeukaryotes in nutrient cycling

Water mixing had important implications on nutrient cycling supported by previous results (Geraldes & Boavida, 2005; Qin et al., 2020). First, core taxa drove the nutrient cycling in both surface and bottom waters. This may be because core taxa maintain nutrient cycling by facilitating the community stability to environmental changes, consistent with a previous study in the soil environment (Jiao et al., 2022b). Second, core taxa facilitated nutrient cycling by maintaining community abundance. Compared with satellite taxa, the absolute abundance of core microeukaryotes was more related to total organic carbon (Table S2). For the bottom waters, chlorophyll‐*a* had a larger effect on the MNC index (Figure 5C,D). The change in chlorophyll‐*a* (as a proxy of phytoplankton biomass) driven by water mixing was more significantly related to the core microeukaryotic absolute abundance in bottom waters. Previous studies showed that water mixing regimes can impact the vertical migration of phytoplankton (Bordet et al., 2017; Lofton et al., 2020), further fueling biogeochemistry in aquatic ecosystems (Wirtz et al., 2022). Our previous study showed that phytoplankton (chlorophyll‐*a*) were closely related to the dynamics of the particulate organic matter and nutrient cycling in the deep waters (Gao et al., 2021). Additionally, the bottom satellite taxa had larger consequences for the ecosystem functioning under the water mixing. Satellite taxa normally included large numbers of rare species. Studies have shown that some rare species can have an over‐proportional role in carbon, nitrogen, and sulfate cycles and are a hidden driver of microbiome function (Musat et al., 2008; Pester et al., 2010; Xue et al., 2017). Due to differentiation strategies, not only rare microbial taxa but also core taxa play an indispensable role in deep‐water nutrient cycling in reservoirs.

### Consequences of water quality monitoring

Our study revealed that water mixing induced by water temperature destabilized the bottom microeukaryotic communities and impacted nutrient cycling. Lake surface water temperature increase induced by climate change will cause mixing‐regime alterations (Woolway et al., 2020), and therefore may have a major impact on emissions of methane and downstream water quality (Bastviken et al., 2004; Carey et al., 2022). From the perspective of reservoir management, reservoir microorganisms should remain stable following environmental changes. Thus, long‐term monitoring is necessary for assessing the effects of changing environmental or anthropogenic factors on reservoir microbial communities. It is worth noting that core taxa played a major role in maintaining community stability and nutrient cycling. These core OTUs composed of a few hundred OTUs, included conditionally abundant and conditionally rare taxa, which can rapidly respond to environmental changes, and were highly representative of entire communities. Concentrating on the core OTUs may facilitate the establishment of references for long‐term ecological monitoring in a reservoir, rapidly and accurately forecasting the ecological consequences of current and future environmental changes, therefore paving the way to better protection of reservoir ecosystems.

In summary, our data showed that microeukaryotic abundance, community composition, and interactions in the surface waters could almost recover to the original level after complete water mixing in a subtropical reservoir. Still, they do not recover in the same way in the bottom waters, perhaps due to hydrological regime change. Particularly, this study demonstrated the important ecological roles of the core taxa in maintaining community stability for both surface and bottom waters. However, the beta diversity of satellite communities only influenced the multi‐nutrient cycling in the bottom waters. Our findings not only contribute to filling the gap in microeukaryotic community responses to the water mixing disturbance of deep subtropical reservoirs but also improve our understanding of the stable mechanism of microbial diversity and the microbial responses to environmental perturbation in reservoir ecosystems.

## AUTHOR CONTRIBUTIONS

**Yuanyuan Xue:** Conceptualization (lead); data curation (lead); formal analysis (lead); funding acquisition (supporting); investigation (supporting); methodology (lead); writing – original draft (lead). **Huihuang Chen:** Data curation (supporting); investigation (lead); writing – review and editing (supporting). **Peng Xiao:** Formal analysis (supporting); writing – review and editing (supporting). **Lei Jin:** Investigation (supporting); methodology (supporting); writing – review and editing (supporting). **Ramiro Logares:** Writing ‐ review and editing (supporting). **Jun Yang:** Conceptualization (lead); funding acquisition (lead); supervision (lead); validation (lead); writing – review and editing (lead).

## CONFLICT OF INTEREST STATEMENT

The authors declare that they have no competing interests.

## Supporting information


**Data S1.** Supporting Information.

## Data Availability

Microeukaryotic sequences are available in the NCBI Sequence Read Archive database under BioProject number PRJNA661101.
