# Supplementary material for: Core taxa drive microeukaryotic community stability of a deep subtropical reservoir after complete mixing
Source: Environ Microbiol Rep. 2023 Sep 9;15(6):769–82. doi: 10.1111/1758-2229.13196 (PMC10667671; doi:10.1111/1758-2229.13196)
Supplement: Supplementary file 1 — Data S1. Supporting Information. [file EMI4-15-769-s001.doc]

**Journal: Environmental Microbiology Reports**

*Supplementary information of the article:*

Core taxa drive microeukaryotic community stability of a deep subtropical reservoir after complete mixing

Yuanyuan Xue^1^, Huihuang Chen^1,2^, Peng Xiao^1^, Lei Jin^1,2^, Ramiro Logares^3^, Jun Yang^1^,*

^1^*Aquatic EcoHealth Group, Fujian Key Laboratory of Watershed Ecology, Key Laboratory of Urban Environment and Health,* *Institute of Urban Environment, Chinese Academy of Sciences, Xiamen 361021, China*

^2^*University of Chinese Academy of Sciences, Beijing 100049, China*

^3^*Institute of Marine Sciences (ICM), CSIC, 08003 Barcelona, Spain*

**Running Title: Microeukaryotic stability after water mixing**

***Corresponding author**

Jun Yang, E-mail address: jyang@iue.ac.cn

**This supplementary information contains:**

- 12 Pages
- 8 Figures
- 2 Table

**Supplementary methods**

Water temperature (WT), pH, dissolved oxygen (DO), turbidity (NTU), electrical conductivity (EC), and oxidation-reduction potential (ORP) down the water column were measured *in situ* at 1 m intervals using a multi-parameter water quality analyzer (Hydrolab DS5, Hach, Loveland, CO, USA). Transparency was determined with a 30 cm Secchi disk. Chlorophyll *a* (Chl-*a*) were measured in triplicate *ex situ* by a PHYTO-PAM Phytoplankton Analyzer (Heinz Walz GmbH, Effeltrich, Germany). Total carbon (TC), total organic carbon (TOC), total nitrogen (TN), ammonium nitrogen (NH_4_-N), nitrate nitrogen (NO_3_-N), nitrite nitrogen (NO_2_-N), total phosphorus (TP), and phosphate phosphorus (PO_4_-P) were determined in the lab using standard procedures (Greenberg et al., 1992).

**Reference**

Greenberg, A., Clesceri, L., Eaton, A. (1992) Standard Methods for the Examination of Water and Wastewater. Washington DC, USA: American Public Health Association.


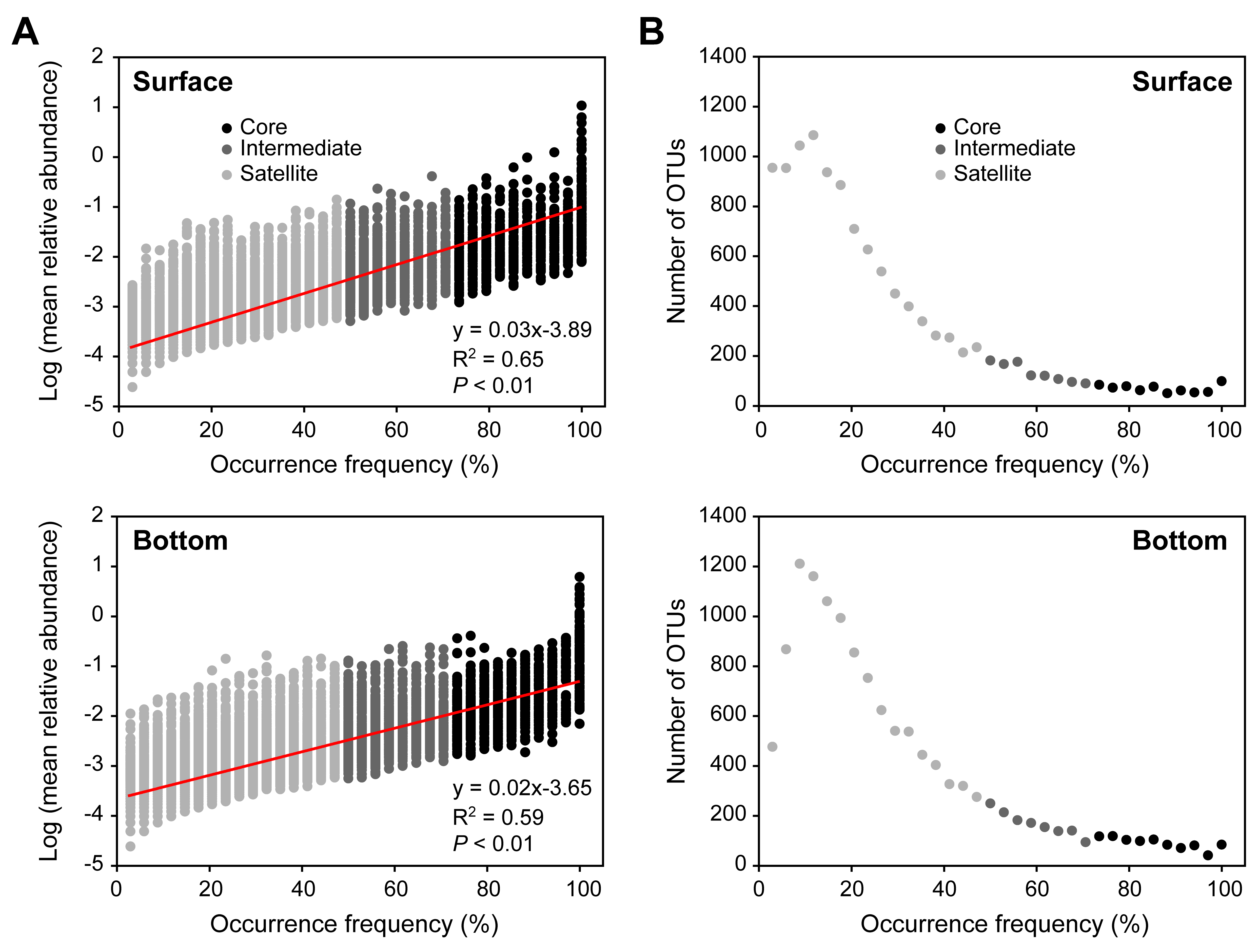


**FIGURE S1** Definition of core, intermediate, and satellite taxa for the surface and bottom waters, respectively. The mean relative abundance of OTUs (**A**) and the number of OTUs (**B**) are shown relative to the OTUs percentage occurrence. Core taxa were defined as the OTUs with an occurrence frequency ≥ 75% in all samples. Satellite taxa were defined as the OTUs with an occurrence frequency < 50% in all samples.


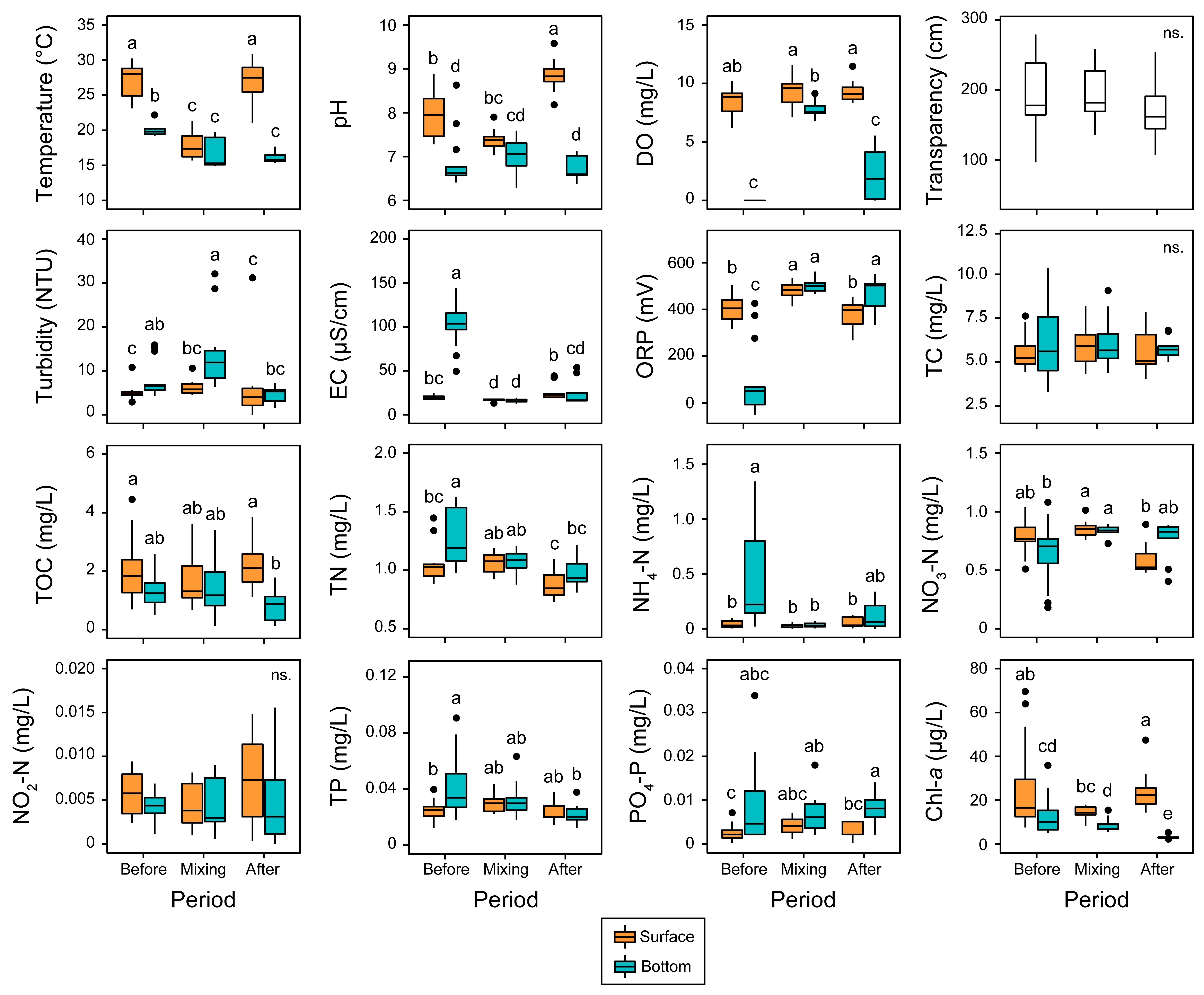


**FIGURE S2** Comparisons of environmental variables at two different water depths across three different periods. Before indicates before the complete mixing (stratification) period; Mixing indicates the complete mixing period; After indicates after the complete mixing (re-stratification) period. DO, EC, ORP, TC, TOC, TN, NH_4_-N, NO_3_-N, NO_2_-N, TP, PO_4_-P and Chl-*a* represent dissolved oxygen, electrical conductivity, oxidation-reduction potential, total carbon, total organic carbon, total nitrogen, ammonium nitrogen, nitrate nitrogen, nitrite nitrogen, total phosphorus, phosphate phosphorus and chlorophyll-*a*, respectively. Different lower-case letters indicate significant differences based on Kruskal-Wallis test at *P* < 0.05 level. ns., non-significant differences. Boxplots show median (black line), 25% and 75% percentiles (box), and range (whiskers); dots represent outliers.


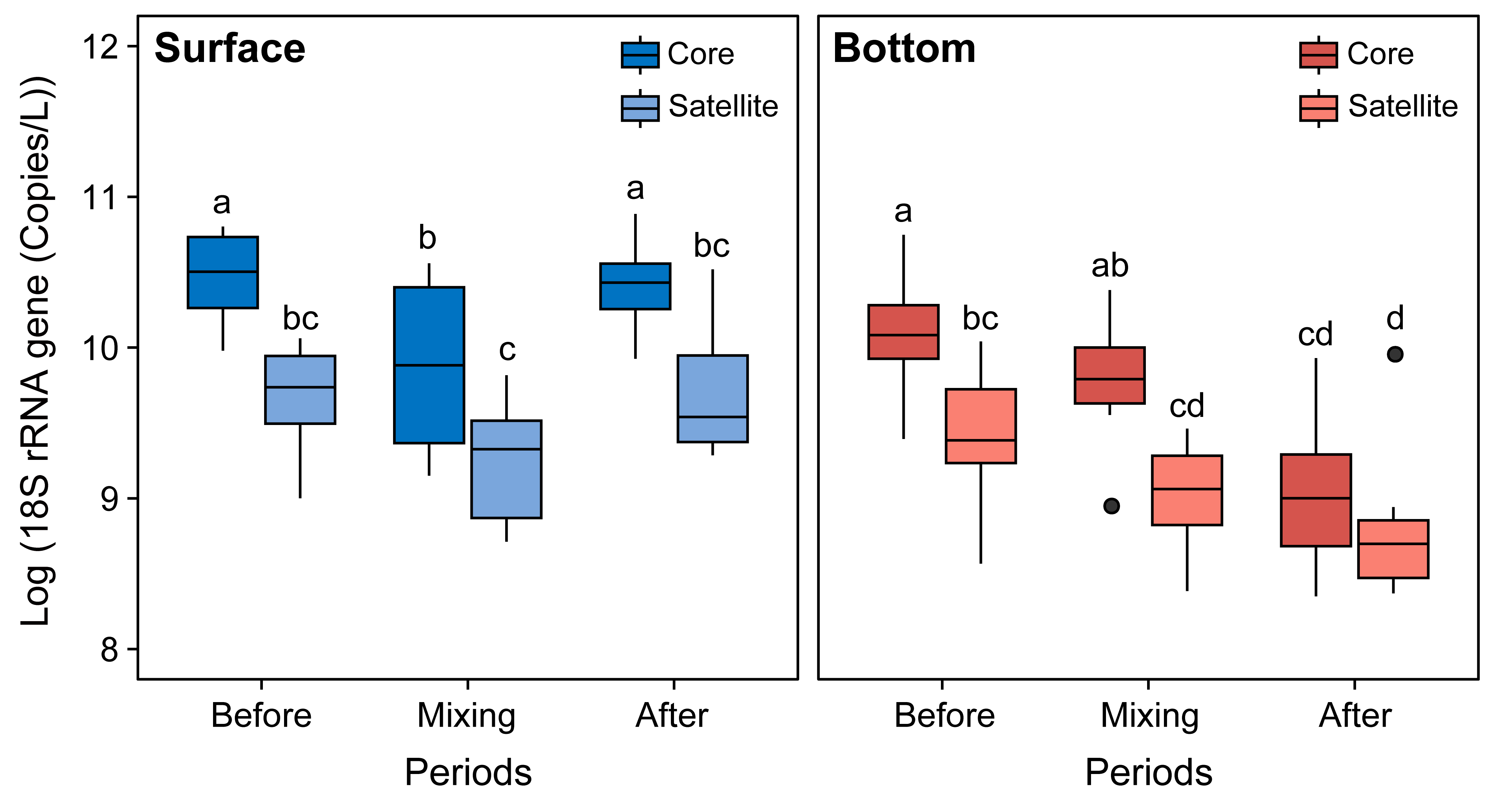


**FIGURE S3** Comparisons of absolute abundances of core and satellite microeukaryotes among different periods for surface and bottom waters, respectively. Different letters represent significant differences between different periods at *P* < 0.05 level using Kruskal-Wallis test. Boxplots indicate median black line, 25% and 75% percentiles box, and range whiskers; dots represent outlier.


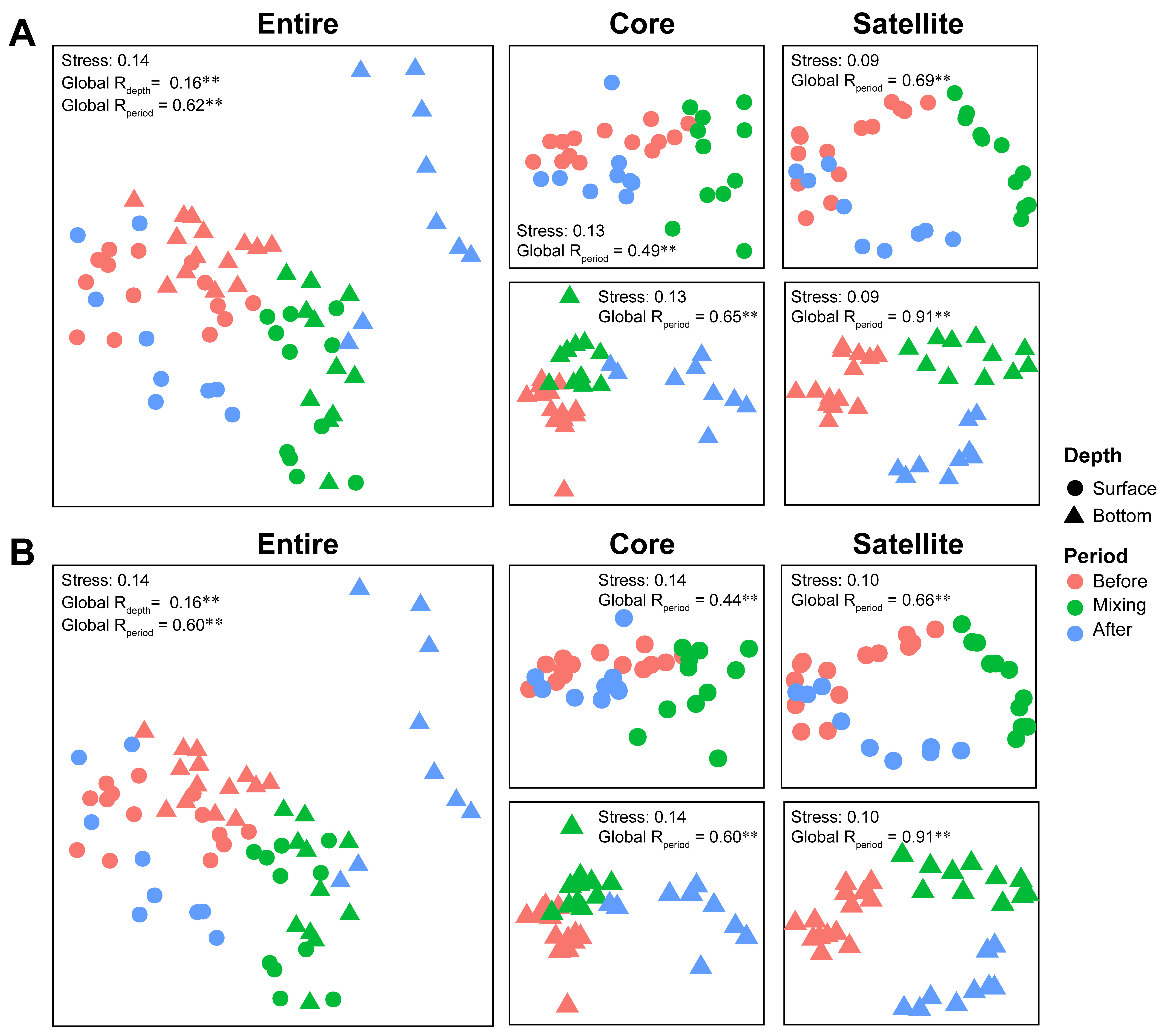


**FIGURE S4** Microbial beta-diversity associated with the three different periods for surface (n = 34 samples) and bottom (n = 34 samples) waters generated by OTUs (**A**) and ASVs (**B**). NMDS based on Bray-Curtis dissimilarity of entire, core, and satellite community compositions showing that water mixing significantly affected microeukaryotic communities, which is examined via the ANOSIM test (***P* < 0.01). Before indicates before the complete mixing (stratification) period; Mixing indicates the complete mixing period; After indicates after the complete mixing (re-stratification) period.


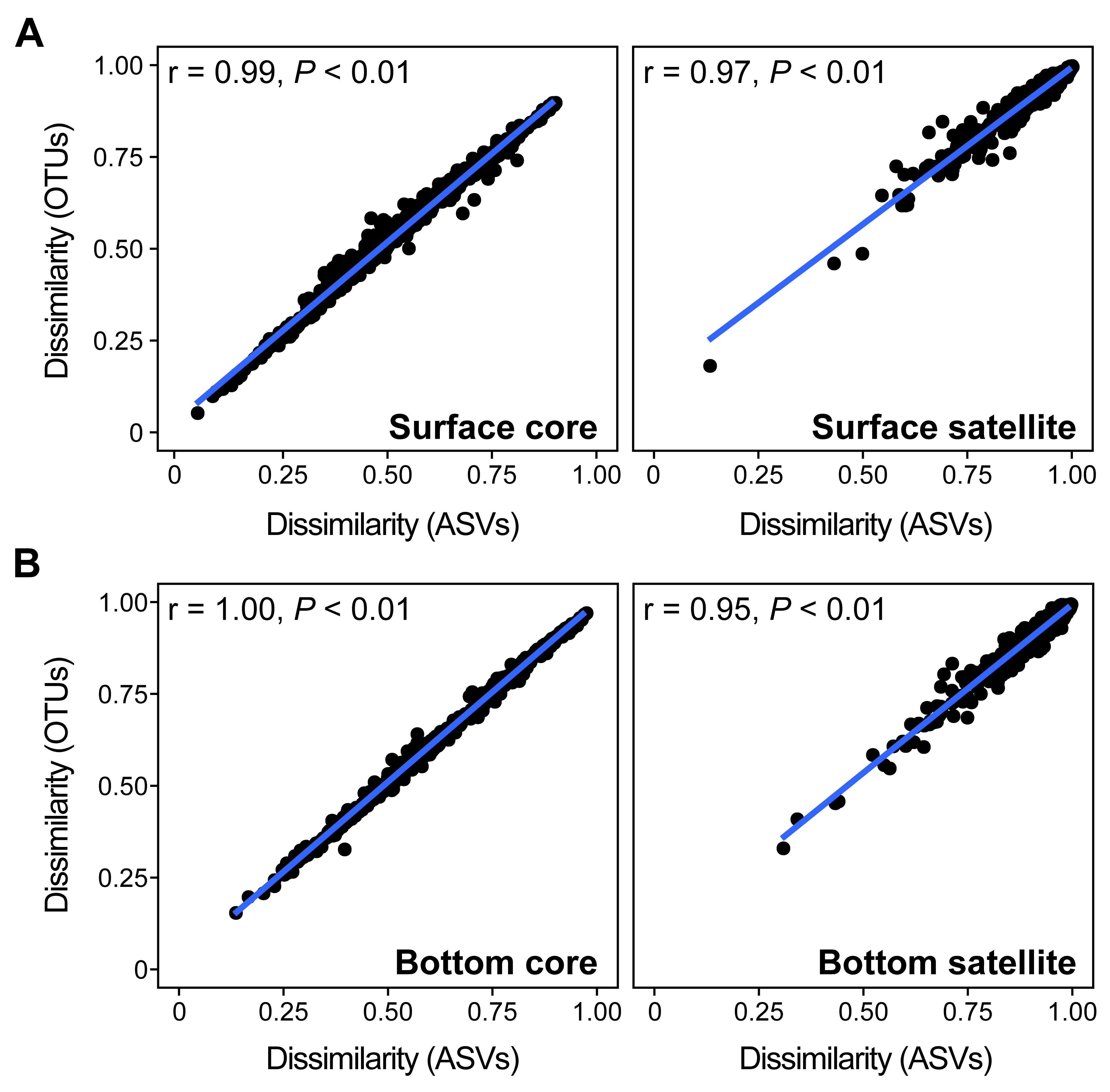


**FIGURE S5** Spearman correlations of pairwise Bray-Curtis dissimilarities of core and satellite taxa generated by the OTUs and ASVs for surface (**A**) and bottom (**B**) waters, respectively.


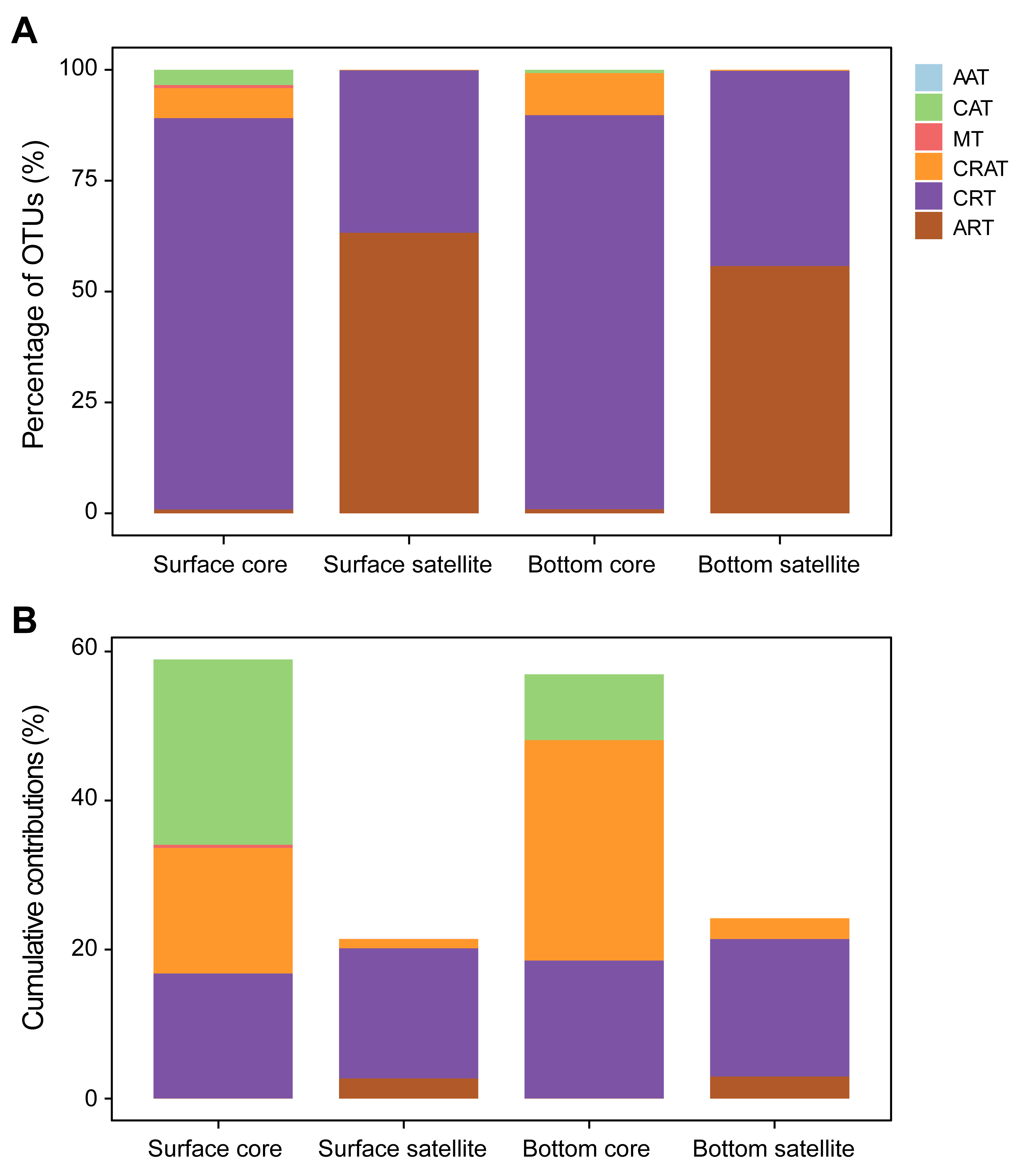


**FIGURE S6** The percentage of different category OTUs and their contributions to entire community dissimilarity for surface and bottom waters. The percentages of abundant and rare OTU numbers within core and satellite taxa (**A**). The cumulative contributions of abundant and rare OTUs within each category to entire community dissimilarity among three periods using similarity percentage (SIMPER) analysis (**B**). AAT, always abundant taxa; CAT, conditionally abundant taxa; MT, moderate taxa; CRAT, conditionally rare and abundant taxa; CRT, conditionally rare taxa; ART, always rare taxa.


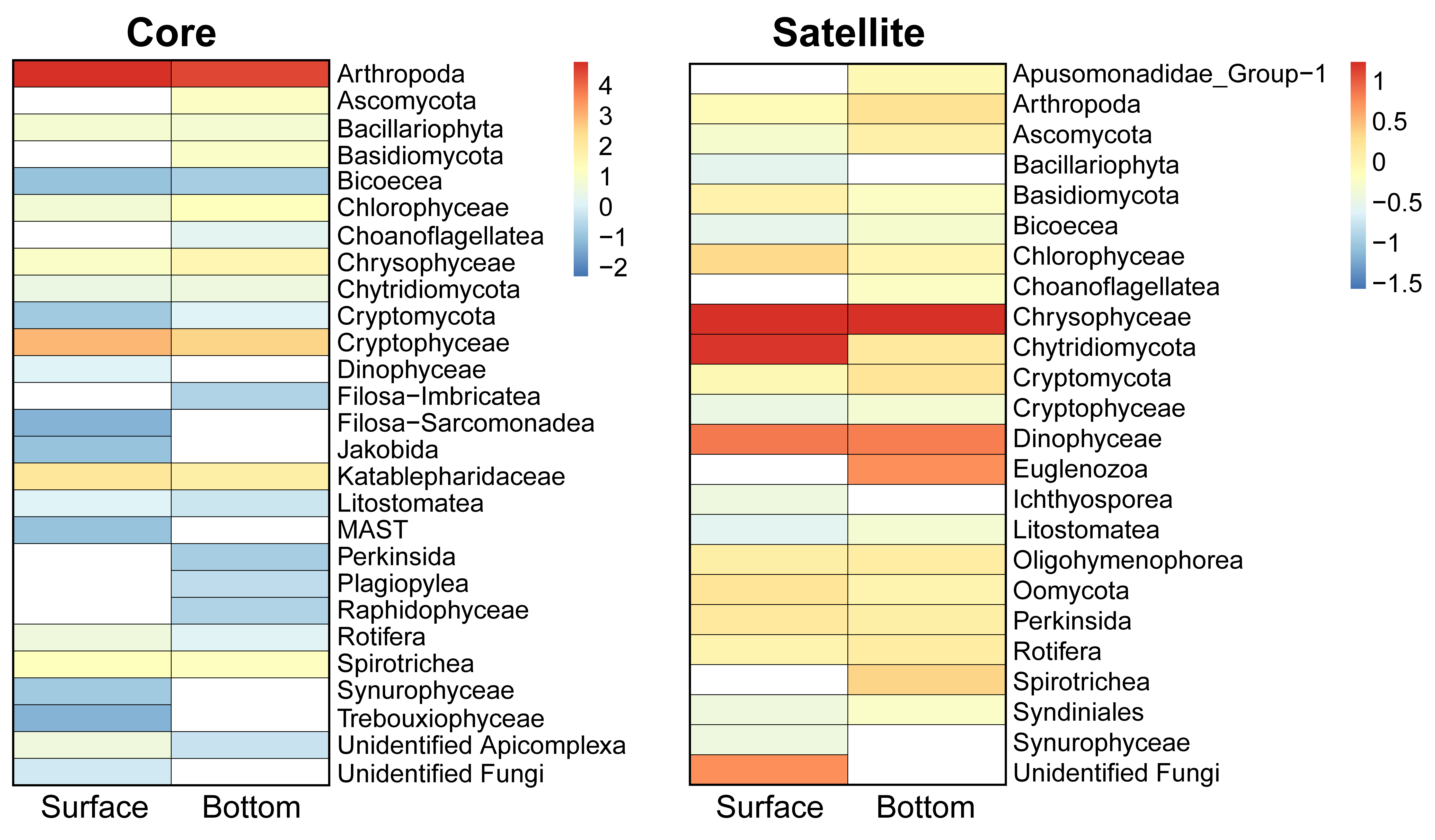


**FIGURE S7** The contributions of top 20 taxonomic groups to the core (or satellite) community dissimilarities across three different periods for surface and bottom waters. Community dissimilarities were calculated by SIMPER analysis. Each cell represents the log_2_ transformed community dissimilarity of each taxonomic group in surface and bottom waters. Blank cells indicate the contributions of taxonomic groups to community dissimilarities are not in the top 20.


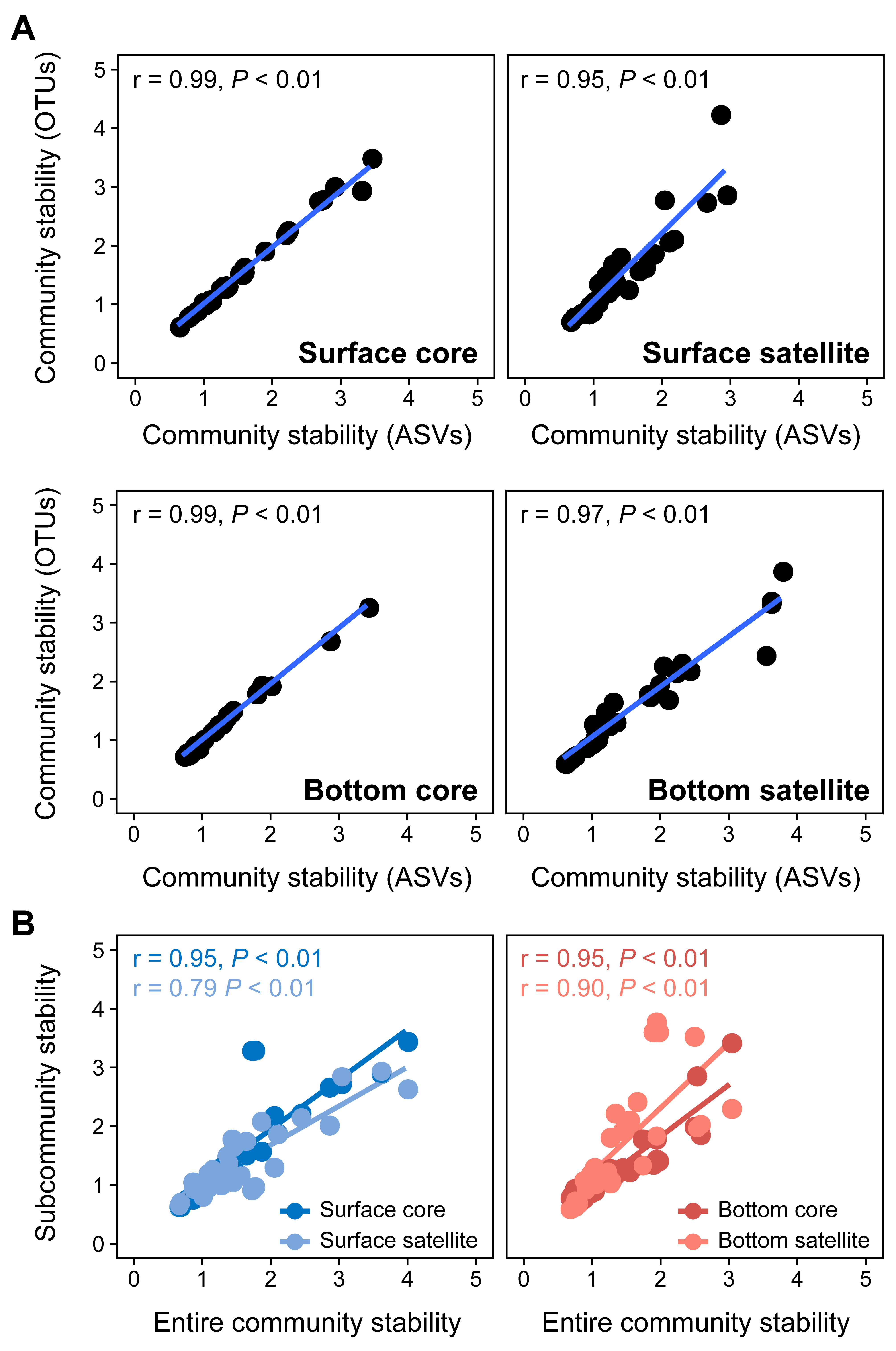


**FIGURE S8** Comparisons of the core and satellite microeukaryotic community stability between OTUs and ASVs, respectively. Community stability was quantified as the inverse coefficient of variation (1/CV) of microbial absolute abundance over each 5-time point window. Relationships between community stability generated by OTUs and ASVs for surface and bottom waters, respectively (**A**). Relationships between core or satellite community stability and the entire community stability for surface and bottom waters generated by ASVs, respectively (**B**).

TABLE S1 Descriptions of the surface and bottom microeukaryotic datasets in Tingxi Reservoir generated by OTUs and ASVs, respectively.

|  | Depth | Category | OTU number | Sequence number |
| --- | --- | --- | --- | --- |
| OTUs | Surface | Core | 605 (5.2%) | 2890036 (70.1%) |
|  |  | Satellite | 9915 (85.0%) | 609622 (14.8%) |
|  |  | Total | 11660 | 4122262 |
|  | Bottom | Core | 782 (6.0%) | 2668246 (64.7%) |
|  |  | Satellite | 10839 (82.9%) | 788023 (19.1%) |
|  |  | Total | 13079 | 4122262 |
| ASVs | Surface | Core | 85 (3.4%) | 2634643 (64.9%) |
|  |  | Satellite | 2315 (91.4%) | 880055 (21.7%) |
|  |  | Total | 2533 | 4060790 |
|  | Bottom | Core | 107 (2.8%) | 2447733 (60.3%) |
|  |  | Satellite | 3526 (92.4%) | 1076126 (26.5%) |
|  |  | Total | 3818 | 4060790 |

TABLE S2 Spearman rank correlations between environmental factors and the absolute abundances of core and satellite microeukaryotes for surface and bottom waters.

| Environmental variables | Surface core | Surface satellite | Bottom core | Surface satellite |
| --- | --- | --- | --- | --- |
| Transparency | -0.127 | -0.163 | 0.167 | 0.140 |
| Water temperature | 0.588** | 0.615** | 0.455** | 0.405* |
| pH | 0.468** | 0.486** | 0.118 | -0.025 |
| Dissolved oxygen | -0.034 | 0.071 | -0.168 | -0.295 |
| Turbidity | -0.124 | -0.173 | 0.336 | 0.252 |
| Electrical conductivity | 0.604** | 0.564** | 0.470** | 0.475** |
| Oxidation reduction potential | -0.583** | -0.676** | -0.389* | -0.390* |
| Total carbon | 0.139 | -0.087 | -0.112 | -0.262 |
| Total organic carbon | 0.561** | 0.439** | 0.463** | 0.309 |
| Total nitrogen | -0.169 | -0.135 | 0.269 | 0.218 |
| Ammonium nitrogen | 0 | 0.165 | 0.113 | 0.282 |
| Nitrate nitrogen | -0.131 | -0.028 | 0.043 | 0.042 |
| Nitrite nitrogen | 0.186 | 0.002 | 0.051 | 0.042 |
| Total phosphorous | 0.010 | 0.088 | 0.274 | 0.226 |
| Phosphate phosphorus | -0.108 | -0.228 | -0.258 | -0.195 |
| Chlorophyll-*a* | 0.325 | 0.411* | 0.646** | 0.494** |

**P* < 0.05, ***P* < 0.01
